# Vitrimer synthesis from recycled polyurethane gylcolysate

**DOI:** 10.3389/fbioe.2023.1209294

**Published:** 2023-07-24

**Authors:** Yu-Hsuan Lin, Yun-Lin Chen-Huang, Alex C.-C. Chang

**Affiliations:** ^1^ Chemical Engineering Department, Feng Chia University, Taichung, Taiwan; ^2^ Green Energy and Biotechnology Industry Research Center, Feng Chia University, Taichung, Taiwan

**Keywords:** vitrimer synthesis, polyurethane waste, glycolysis, experimental design, circulatory economy

## Abstract

Polyurethanes and plastics have become ubiquitous in modern society, finding use in a wide variety of applications such as clothing, automobiles, and shoes. While these materials provide numerous benefits to human life, their persistence in the environment has caused ecological imbalances. Therefore, new processes are needed to make these materials more sustainable and re-usable. In 2011, Ludwik Leibler introduced a new class of covalent adaptable network (CAN) polymers called Vitrimers. Vitrimers possess self-repairing properties and are capable of being reprocessed due to dynamic exchange or breaking/recombination of covalent bonds, similar to thermoset materials. This study explores the synthesis of Vitrimers using waste polyurethane or plastics as feedstock. The raw materials were glycolysed to obtain the glycolysate, which was then used as a reagent for the Vitrimers synthesis. The main objective of this study was to achieve the maximum self-repairable rate of the prepared sample. The Taguchi orthogonal analysis was employed to guide the experiments. The optimized experimental conditions for polyurethane glycolysis were determined to be under ethylene glycol and catalyzed by sodium hydroxide at 180°C for 1 h, resulting in the highest hydroxyl concentration in the glycolysate. In the second stage of the experiment, the ratio of hexamethylene diisocyanate (HDI) to solvent was set to 2, HDI trimer to solvent was 2, and PGE/glycolysate was 0.5, with equal amounts of PEG and glycolysate used as the solvent. The reaction was carried out at 80°C for 1 h, achieving a self-repair ability of 47.5% in the prepared sample. The results of this study show that waste polyurethane or plastics can be effectively recycled and transformed into vitrimers with self-repairing properties. The use of glycolysis as a feedstock is a promising method for the sustainable recycling of polyurethane waste. The Taguchi orthogonal analysis is an effective approach for optimizing experimental conditions and improving the reproducibility of the results.

## Highlights

• Vitrimer was synthesized by polyurethane glycolysate.
• Prepared vitrimer was self-reparable.
• Polyurethane and polymer glycolysation were optimized by the experimental design.
• Taguchi experimental design method was used in this study.

## 1 Introduction

In 2011, French researcher Ludwik Leibler reported on a new class of covalently associated networks (CANs), which he named vitrimers. When heated, vitrimers behave like a viscoelastic liquid, but when exposed to solvent they will only show a limited soluble fraction, related to defects and unattached chains. Vitrimers derive these properties from a covalent molecular network that can change its topology through molecular rearrangements, while preserving the total number of bonds in the network, i.e., the network connectivity. When a vitrimer is heated above its glass (or melting) temperature, molecular rearrangements can take place and the topology of the network fluctuates. Thus, network segments can diffuse even though the network preserves its connectivity while spanning the volume of the whole sample. In terms of material properties, the permanent nature of the network enhances the resistance to polymer wear processes such as dissolution, creep, or solvent stress cracking. The dynamic nature of the network facilitates processing and reprocessing and brings recycling possibilities despite the presence of permanent covalent cross-links (Winne et al., 2019). The main concept is to design a material that can repair itself after damage. The synthesis process involves only simple chemistry and basic physical principles, making it relatively easy to carry out. Additionally, alcohol polymers such as polyethylene glycol can be used as one of the main sources. After years of experimentation, it has been found that polyols in polyurethane glycolysate could be potential candidates for vitrimer synthesis. This also creates future opportunities for the recycling industry (Liu et al., 2023; Wu et al., 2023).

Vitrimers are a class of plastic materials that are derived from thermosetting polymers (also known as thermosetting plastics), so they are very similar to them. They consist of a covalent network of molecules that can change their internal geometry through thermally activated bond exchange reactions. At high temperatures, they flow like viscoelastic liquids, while at low temperatures, the bond exchange reactions slow down (freeze) and they behave like a hybrid of glass-ceramics and thermosets.

Poly (ethylene terephthalate) (PET) is one of the most used polymers worldwide because of its set of properties, including good mechanical strength, thermal and chemical stability, processability, and low permeability to gases. For this reason, its applications range from food and beverage packaging (mainly bottles and trays) to vehicle parts, electronic instruments, and fibres. Its large diffusion poses challenges for the end-of-life management of PET household waste, as well as the dispersion of microparticles in water, mainly derived from PET fibers. There is now a well-established recycling process for PET bottles. However, recycling PET from food trays (tPET) remains challenging due to its lower molecular weight and the possible presence of other polymers resulting from the use of multi-layered trays. In this work, scrap PET trays have been chemically modified through melt reaction processing to form CANs, thereby enhancing their properties (Fabrizio et al., 2023).

Polyurethane (PU) thermosets are the sixth-largest class of synthetic polymers and have found widespread use in elastomers, foams, adhesives, coatings, and structural components. The desirable mechanical properties of PU thermosets come from the combination of hard and soft domains. Hard domains are formed from urethane groups capable of forming hydrogen bonds, which provide stiffness and toughness, while soft domains from aliphatic monomers provide flexibility to the material. However, recycling the covalently crosslinked network of PU thermosets is exceptionally difficult. Although mechanical milling methods have been used to downcycle PU thermosets into fillers to reinforce adhesive composites, chemical recycling of PU thermosets remains a challenge (Li et al., 2021).

Due to its chemical versatility, PU has become a promising and popular class of material in households and industries. However, the usage of this material generates a lot of affiliated waste, and a new approach to address this issue is needed. Jian Li originally proposed a novel PU vitrimer concept with excellent mechanical properties, improved solvent resistance, and excellent recyclability (Li et al., 2022).

PU and PET are mainly recyclable polymers. Polyurethane recycling is a topic with which a number of research teams are dealing with. The leading techniques adopted for PU recycling are glycolysis, ammonolysis, hydrolysis, and pyrolysis. Industrial recycling largely involves ammonolysis. Glycolysis is among the simplest and most effective methods of polyurethane recycling. During this process, the waste polyurethane foams are crushed and reacted with a glycol at ambient conditions of temperatures to produce a new type of recycled polyol. These are further separated to react with isocyanate to produce new polyurethanes. The newly obtained polyurethane possesses almost all the properties of the previous one and can be thus used for all its applications (Reghunadhan et al., 2020).

Some background on isocyanate chemistry is reviewed to describe the chemical properties of isocyanates, specifically the addition reaction between isocyanate groups and active hydrogen-containing compounds, and the thermal reversibility of their products. These properties provide opportunities for chemical reformulation and recycling (Delebecq et al., 2013).

The vitrimer-thermoset concept represents an important contribution to improving the durability and recyclability of thermoset systems. The associative dynamic covalent adaptive network (ADCAN) concept, based on thermoset vitrimers, exhibits high levels of stress relaxation, self-healing, and shape memory properties. In this vitrimer, the dynamically fixed crosslink density allows reprocessing of the material without loss of macroscopic properties based on temperature-dependent association exchange. The overall reprocessing can be achieved by pure associative bond exchange, governed by its kinetic profile and tunable by the catalyst used (Krishnakumar et al., 2020).

Francesco Gamardella et al. used dibutyltin dilaurate (DBTDL) as a catalyst to develop different polythiourethanes (poly-thiourethane) and recyclable thermosetting materials. Although aromatic diisocyanates can be synthesized into polythiourethane under different catalysts, the resulting material is not recyclable. FTIR and thermomechanical tests showed that the material synthesized with DBTDL as a catalyst maintains a good cross-linking structure and mechanical properties. The recovered materials also demonstrate the potential for synthesizing polymeric materials through structural rearrangement (Gamardella et al., 2020a; Gamardella et al., 2020b).

Liu et al. (2020) attempted to combine polyvinyl alcohol and ε-caprolactone compounds with disulfide-containing polyurethane synthesized from isolated isocyanates using the grafting method. The material was able to self-heal at 2.5 MPa and 90°C for 1 h, achieving a self-healing rate of 94% after being destroyed in experiments. This suggests a potential route for developing a PU recycling method (Rong et al., 2021).

Currently, the development of recyclable polymers has become a very important issue. Considerable attention has also been given to the development of high-strength, durable, and chemically resistant materials that are similar to conventional thermosetting polymers but have self-healing abilities. Vitrimers are a promising candidate for achieving these goals (Meyer et al., 2021).

Shi et al. (2020) demonstrated the post-processing of recycled polyurethane with a dynamic covalent polymer network using a solvent-free and environmentally friendly method. This approach not only provides self-healing capabilities to the material but also reduces the production cost without affecting its mechanical properties.

Today, these materials are no longer just an academic concept; they are produced industrially and are ubiquitous in everyday life due to their low cost and desirable physical properties. The polymer chains in vitrimers have permanent networks linked by dynamic covalent bonds, allowing the network to change its internal structure while maintaining a constant number of chemical bonds at all temperatures. Vitrimers are characterized by high mechanical properties and easy processing, and they have been successfully produced from academic laboratories to industry (Van Zee and Nicolaÿ, 2020/05).

**FIGURE 1:**
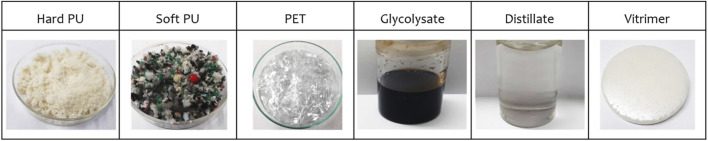
The changes in the material’s appearance in this study.

In this study, waste polyurethane (hard-PU or soft-PU) or waste plastics (PET) were used as raw materials for the synthesis of vitrimers. The Raw materials were glycolyzed to have the glycolysate, the glycolysate was double distillate to have the distillate, and the distillate was used as the material for vitrimer synthesis. The change of the material’s appearance were shown in Figure 1. The vitrimers were obtained from recycled polyols after glycolyzed, with the aim of finding a way to reuse existing waste materials. The main conditions for the glycolysation reaction, such as material sources, solvents, catalysts, and reaction temperature, were investigated, and the glycolysation solution with the highest hydroxyl concentration was used as the raw material for the synthesis of vitrimers. The weight ratio of the obtained glycolysation solution to HDI, HDI trimer, polyethylene glycol, and reaction time were considered as the main conditions for the synthesis of vitrimers, with the goal of achieving maximum self-repair rate. The Taguchi experimental design method was employed to efficiently determine the optimal degradation and synthesis parameters.

## 2 Materials and methods

### 2.1 Glycolysis of the feedstock

In this study, waste materials from three different sources, including high-density polyurethane (hard PU), low-density polyurethane (soft PU), and polyethylene terephthalate (PET, No. 1 plastic waste plastic bottles), were provided by waste recyclers in central and southern Taiwan. The collected materials were crushed and mixed with catalyst [NaOH, Ca(OH)2, or (CH3COO)2Zn, 2% by weight of the feedstock] and added to a thermal-static reactor with a solvent [ethylene glycol (C2H6O2), diethylene glycol (C4H10O3), or glycerol (C3H8O3); same weight as the feedstock] at the boiling point of the solvent. After the feedstock was fully dissolved into the solvent, the solution was kept at the reaction temperature for an hour to obtain the waste glycolysate.

The Taguchi L9 (3^3^) orthogonal array was applied as the experimental design model to determine the highest hydroxyl concentration of the glycolysate. The feedstock (parameter A, P-A), catalyst (parameter B, P-B), and reaction solvent (parameter C, P-C) were the design factors for the glycolysation experiments. Three different levels were selected for each design factor. The factors and levels for the Taguchi Analysis Array used in this study are shown in Table 1.

**TABLE 1:** Experimental design for feedstock glycolysis.

Levels Factors	P-A	P-B	P-C
	Waste	Catalyst	Solvent
Level – 1	Hard PU	NaOH	Ethylene Glycol
Level – 2	Soft PU	Ca(OH)2	Diethylene glycol
Level – 3	PET	(CH3COO)2Zn	Glycerol

The optimized glycolysate was purified by removing the catalyst and double distillation. The collected distillate was used as the precursor for the vitrimer synthesis in the second stage of the experiment.

### 2.2 Vitrimer synthesis

Solvent-free methods were adopted to prepare the vitrimer. During the reaction, 10 g of polyethylene glycol (PEG, 25322-68-3, SHOWA, 99%), prepared glycolysate, Hexamethylene diisocyanate [HDI, 822-06-0, Tokyo Chemical Industry (TCI), >98.0%], and 1,4-Diazabicyclon [2.2.2]octane (DABCO, 280-57-9, Alfa Aesar, 98%, served as the catalyst; 0.5% by weight of the total alcohol weight) were mixed at the designated ratio at the reaction temperature. The Bisphenol S [BPS, 80-09-1, Tokyo Chemical Industry (TCI), >98.0%, served as the hardening promoter; 25% by weight of the total alcohol weight] and HDI trimer were then added to the mother solution at the designated ratio. The reaction was held for 1 h at 85°C (Yan et al., 2017). The prepared sample was then transferred to the silicone gel mold to form the vitrimer. Self-healing tests, FTIR analysis, and tensile tests were performed.

The Taguchi L9 (4^3^) orthogonal array was applied as the experimental design model to determine the highest hydroxyl concentration of the glycolysate. The HDI/Alcohol (parameter A, P-A), HDI-Trimer/Alcohol (parameter B, P-B), PEG/Glycolysate (parameter C, P-C), and Time (min) (parameter D, P-D) were considered as the design factors for preparing the vitrimer. Three different levels were selected for each design factor. The factors and levels for the Taguchi Analysis Array used in this study are presented in Table 2. The optimized condition for the self-healing ability will be selected, along with some other properties as references.

**TABLE 2:** Experimental design for vitrimer synthesis from glycolysate.

Levels Factors	P-A	P-B	P-C	P-D
	HDI/alcohol	HDI-trimer/alcohol	PEG/glycolysate	Time (min)
Level – 1	1	1	0.5	60
Level – 2	2	2	1	70
Level – 3	3	3	2	80

Note: Alcohol: total weight of PEG and glycolysate.

### 2.3 Hydroxyl values of glycolysate

The hydroxyl value is a key factor in determining the results of the glycolyzation process. The acetic anhydride-p-toluenesulfonic acid-ethyl acetate assay method was used to determine the hydroxyl value of the glycolyzation product. In this analysis, ethyl acetate was used as a solvent, p-toluenesulfonic acid as a catalyst, and acetic anhydride as an acetylation reagent. The acetyl group provided the acid, and the glycolyzation product provided the hydroxyl group for the esterification reaction. Finally, the excess acetic anhydride was hydrolyzed into acid by a pyridine aqueous solution and titrated with 0.5 M potassium hydroxide. The acid produced by the excess acetic anhydride can be used to predict the hydroxyl value contained in the glycolyzation product. The hydroxyl value is expressed in milligrams of potassium hydroxide per gram of the weighted sample, and the unit is mgKOH/g.

### 2.4 Self-healing test of the prepared vitrimer

The goal of this research is to use polyols obtained from glycolysate to replace PEG in vitrimer synthesis, and to synthesize new materials through the reaction mechanism of microcrystalline copolymerized polymer structures to create new products with higher-value vitrimers (Shi et al., 2019).

Vitrimers rely on the covalent adaptive network (CANs) and chemical bond breaking and re-linking so that it has the characteristics of self-healing, recyclability, and solderability. When it is not heated, its performance will be like a thermosetting polymer, because there are bonds between their molecular chains. After being heated, the bonds will break and then regenerate new bonds, so they will behave more like a thermoplastic polymer for a short time. The covalent adaptive network is divided into two types, namely, the dissociation type and the binding type. The dissociation type CANs will break the bond first and then generate new bonds elsewhere, and the binding type CANs will only form new chemical bonds. The original chemical bond will be broken. The advantage of the dissociation type is that the viscosity of our product will be lower and the fluidity will be higher during the heating process, so we can do better processing. The advantage of the combined CANs is that although the fluidity is not high during the heating process, it is more stable than dissociated CANs, and in this study, we used dissociated CANs (Denissen et al., 2016).

The prepared vitrimer samples were shaped by a round-shaped template with 5 cm in diameter and 1 cm in thickness. The self-healing test was applied by using a utility knife to cut through the sample (1 cm in depth) with a 2 cm in length scar of the samples respectively. The cut sample was then kept in an oven at 60°C for 15 min. After the testing time, the length of the scar was measured to observe their self-healing ability. The sample with the shortest scar will be the one with the best self-healing ability. The optimized sample preparation condition will also be determined by calculating the S/N ratio by the Taguchi Analysis processes.

### 2.5 Functional group analysis by FTIR

The functional groups of the prepared samples were determined using FTIR analysis. The prepared sample was placed onto the attenuated total reflection (ATR, PIKE Technologies MIRacle Single Reflection ATR) module and the module was loaded into the FTIR bench (Shimadzu Prestige 21 with TDGS detector). The measured spectral range was 4,000 to 400 cm^−1^, to understand the changing behavior of functional groups.

### 2.6 Tensile test of the prepared vitrimer

The tensile testing was performed according to the ASTM D638 standard. The test results were obtained based on the specified tensile speed and load applied to the test piece. In each test, it was performed at room temperature (25°C) with a stretching rate of 40 mm/min. The sample size of each test is 7 cm*2 cm*0.5 cm. However, in actual use, the loads acting on components or structures may vary widely in terms of deformation rates. Owing to the viscoelastic nature of polymers, the actual mechanical properties generated at varying strain rates may differ from those measured using standard test pieces. Hence, the characteristic values obtained by the tensile test method have little reference value for component design but provide a reliable database for material comparison.

## 3 Results and discussion

### 3.1 The hydroxyl value of the glycolysate

**FIGURE 2:**
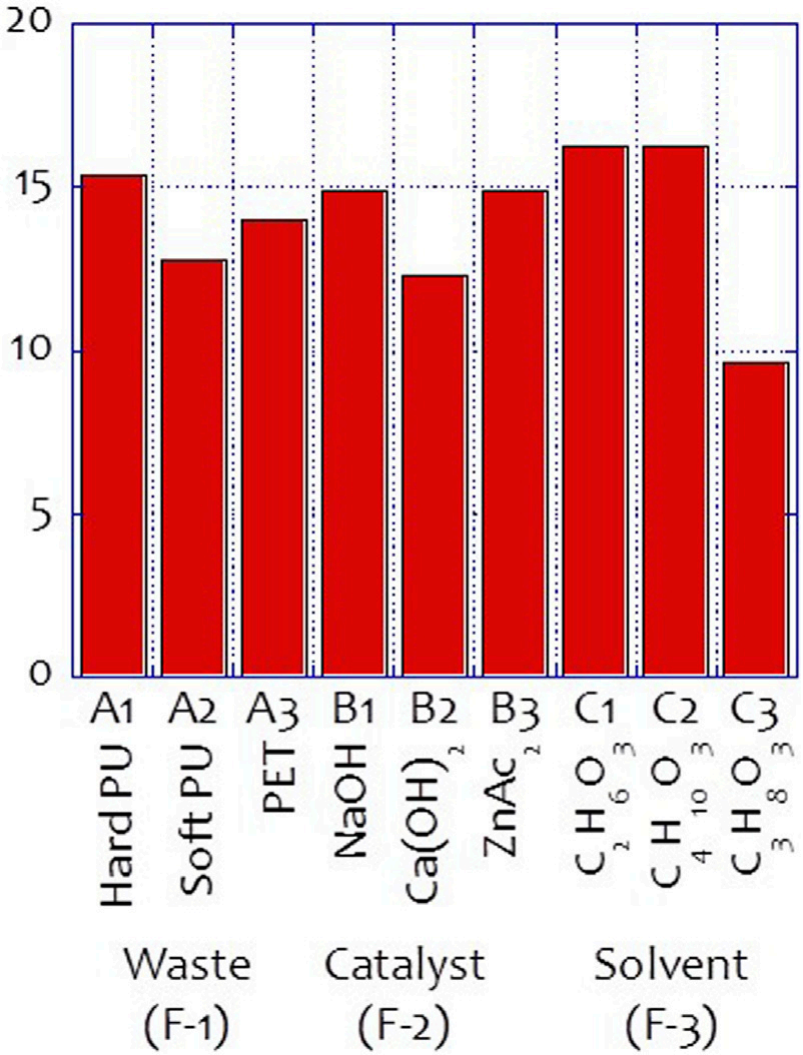
Hydroxyl values signal to noise analysis of the glycolysate.

The hydroxyl value of the glycolysate distillate was determined, and the results are listed in Table 3. Figure 2 also shows the results of the signal-to-noise (S/N) ratio analysis of the waste glycolysation in the Taguchi Analysis. Based on these analysis results, the optimized conditions for achieving the highest hydroxyl value of the glycolysate were determined to be hard PU glycolysation using ethylene glycol as the solvent and sodium hydroxide as the catalyst. The differences between the highest and lowest S/N ratio also revealed the importance of the design factors. Among these factors, the usage of the solvent in the glycolysation was the most important, and the catalyst was the second most important factor in achieving the highest hydroxyl value of the glycolysate at O1-L9-1 (51.91 mgKOH/g of the sample). After adapting the optimized conditions, the optimized hydroxyl value (O1-L9-Opt, 51.91 mgKOH/g of the sample) was also achieved.

**TABLE 3:** Experimental results of hydroxyl concentration of glycolysate.

Experimental design-1	L9 orthogonal array			OHV (mgKOH/g)		Result
	Material	Catalyst	Solvent	Test_1	Test_2	S/N
O1-L9-1	Hard PU	NaOH	C2H6O2	388.72	399.44	51.91
O1-L9-2	Hard PU	Ca(OH)2	C4H10O3	296.63	294.73	49.42
O1-L9-3	Hard PU	(CH3COO)2Zn	C3H8O3	80.57	64.83	36.94
O1-L9-4	Soft PU	NaOH	C4H10O3	296.30	275.20	49.08
O1-L9-5	Soft PU	Ca(OH)2	C3H8O3	5.78	12.51	16.64
O1-L9-6	Soft PU	(CH3COO)2Zn	C2H6O2	289.77	299.39	49.38
O1-L9-7	PET	NaOH	C3H8O3	48.52	43.67	33.20
O1-L9-8	PET	Ca(OH)2	C2H6O2	175.36	178.28	44.95
O1-L9-9	PET	(CH3COO)2Zn	C4H10O3	241.63	238.65	47.61

Note: OHV means the hydroxyl values of the glycolysate.

After obtaining the optimized conditions for the highest hydroxyl value of the glycolysate, the hard PU glycolysation using ethylene glycol was prepared for vitrimer synthesis. The glycolysates were analyzed after the freshly prepared solution was distilled to obtain a translucent clear solution, which was suitable for vitrimer synthesis in the next step.

### 3.2 Self-healing test of the vitrimer

**FIGURE 3:**
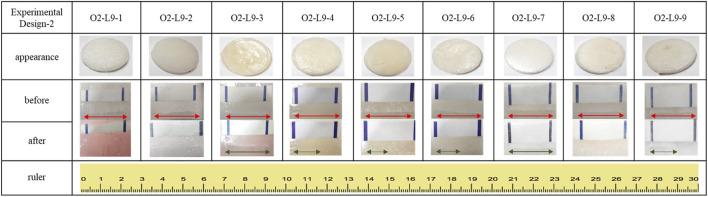
The appearance of the prepared vitrimers in the self-healing ability test.

**FIGURE 4:**
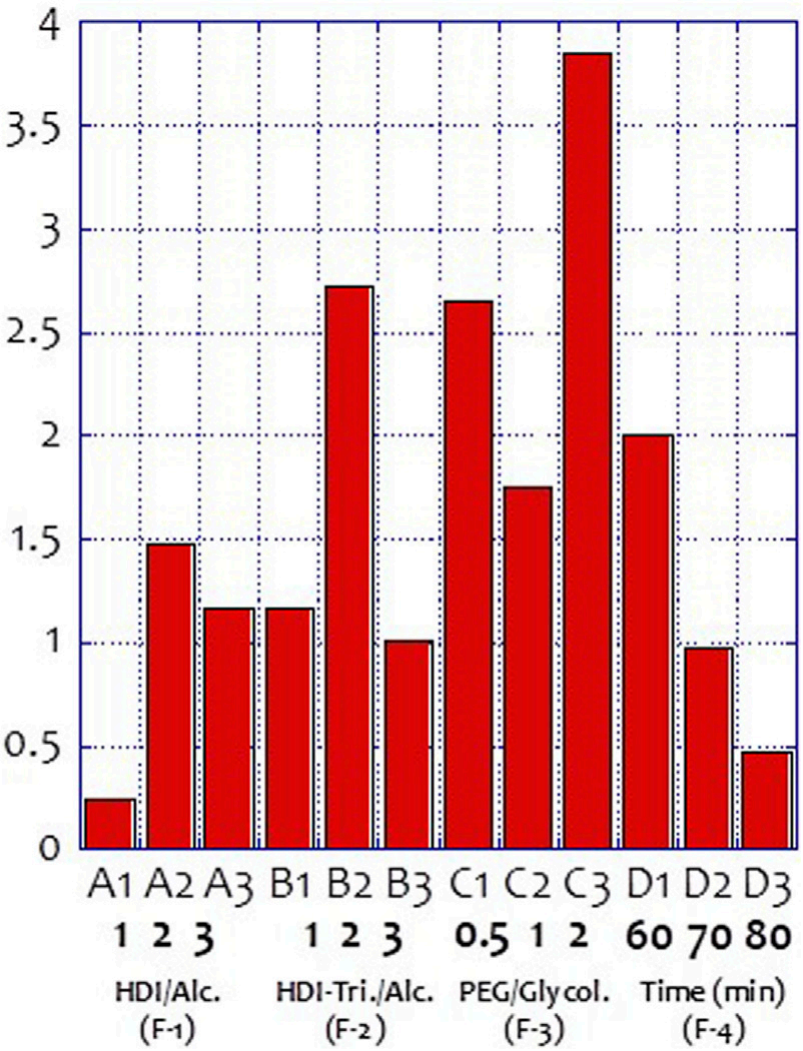
Self-healing ability signal to noise analysis of the prepared vitrimers.

Figure 3 and Table 4 show the self-healing ability test of the prepared vitrimer. The vitrimer was formed in a silicone template with a diameter of 5.0 cm and a thickness of 1.0 cm. After curing, a 2.0 cm cut was made, and the sample was kept in a 60°C oven for 15 min. The length of the cut was then measured again. If the cut was only 1.05 cm, the self-healing rate was defined as 47.5%. The signal-to-noise analysis was then conducted to find the optimized conditions for the highest recovery rate. Figure 4 shows the highest self-healing rate was obtained for the sample prepared at 60°C, using HDI/total alcohol weight (the weight of PEG and glycolysate) as the design factor with the highest importance, and the presence of a suitable amount of HDI as the second most important factor for the self-healing rate.

**TABLE 4:** Experimental results of self-healing levels or prepared vitrimers.

Experimental design-2	Result	S/N
	Self-healing test (%)	
O2-L9-1	100.0	9.54
O2-L9-2	100.0	9.54
O2-L9-3	4.8	16.90
O2-L9-4	47.8	3.14
O2-L9-5	61.9	5.38
O2-L9-6	57.8	4.78
O2-L9-7	2.3	23.13
O2-L9-8	100.0	9.54
O2-L9-9	47.5	3.08

### 3.3 FTIR analysis of the feedstock and prepared samples

**FIGURE 5:**
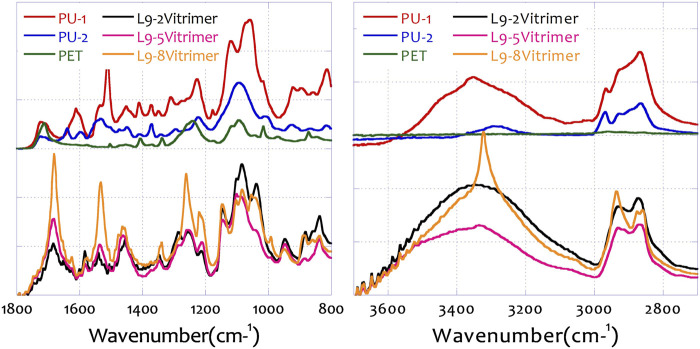
The FTIR results of the feedstocks and the prepared vitrimers.

Figure 5 shows the FTIR analysis of the feedstocks (hard PU, PU-1; soft PU, PU-2; and PET) and the prepared vitrimers. These selected samples, L2-2-vitrimer, L2-5-vitrimer, and L2-8-vitrimer, were the synthesized vitrimers with the designated preparation design coefficients. PU1 and PU2 behaved very similarly to each other, as they were both polyurethanes with different levels of polymerization. In the feedstocks, NH stretching of the amine salt structure was found at the wavelength of 3,361, 2,863, and 2,858 cm^−1^. At the other end of the spectrum with lower wavenumbers, N-O stretching (nitro compound, 1,516 cm^−1^), C-O stretching (vinyl ether, 1,222 cm^−1^), C-O stretching (secondary alcohol, 1,097 cm^−1^), and S=O stretching (sulfoxide, 1,056 cm^−1^) were also found to confirm the structure.

After the vitrimer is prepared, O-H stretching (carboxylic acid, 3,283 cm^−1^), O-H stretching (alcohol, 2,933 cm^−1^), N-O stretching (nitro compound, 1,516 cm^−1^), C-O stretching (vinyl ether, 1,222 cm^−1^), C-O stretching (secondary alcohol, 1,097 cm^−1^), and S=O stretching (sulfoxide, 1,056 cm^−1^) can also be detected in the prepared vitrimers (George Socrates, 2004).

Therefore, in this research work, polyurethane containing amine groups or amine salts in the main structure was successfully treated with polyol-modified glycolysate to form relatively stable covalent bond structures. Different vitrimer synthesis formulas led to the same structure, indicating that the research team was able to transform waste polymer materials into self-healing polymers with higher value.

### 3.4 Tensile testing

**FIGURE 6:**
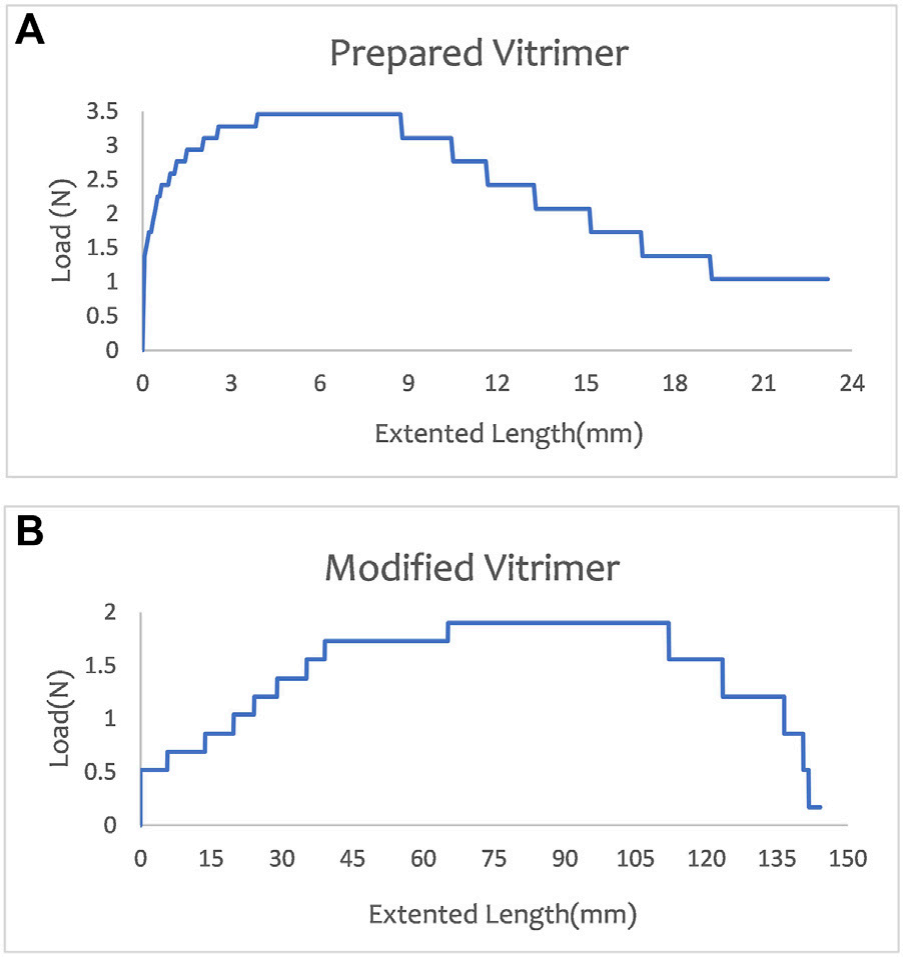
The tensile test results **(A)** Prepared Vitrimer **(B)** Modified Vitrimer.

In tensile testing, load and elongation are measured using testing equipment such as load gauges and displacement gauges. The load and elongation data can then be plotted into a load-elongation curve, which can be analyzed from several aspects: (a) Material strength, which is the ultimate strength of the material and reflects the maximum load that the material can hold; (b) Extensibility of the material, which is the slope of the load-elongation curve and reflects the degree of deformation of the material when it is stretched; (c) Toughness of the material, which is the area under the load-elongation curve and reflects the energy absorbed by the material when it is stretched; (d) Material fracture, which occurs when the elongation of the sample reaches a certain level, causing the load to suddenly drop. The overall dimensions for tensile test specimens are decided as per ASTM D638 standard (standard for tensile testing for vitrimers) (Garg and Bhattacharya, 2017). Figure 6 shows all of the results. The load and elongation analysis is an important part of tensile testing and helps us better understand the properties and behavior of materials.

According to the tensile test results under the original experimental conditions, the sample reached a maximum load of 3.4 N and a maximum elongation of 3.9 mm. The load began to decrease and the sample broke when the elongation reached 8.717 mm. After modifying the preparation conditions, the maximum load of the sample was 1.9 N, and its maximum elongation was 65.222 mm. The fracture phenomenon began to occur after the elongation was 112.03 mm. Therefore, the ductility and toughness of the material were improved by modifying the recipe to prepare the sample.

## 4 Conclusion and suggestions

This research has successfully glycolysed waste polyurethane foam to synthesize vitrimer. The results of this study also demonstrate the feasibility of using waste polyurethane foam as a raw material to synthesize vitrimer, which has potential applications in various fields such as coatings, adhesives, and recyclable materials. The Taguchi experimental design method proved to be a useful tool for optimizing the conditions of both waste glycolysation and vitrimer synthesis, leading to the successful production of high-quality vitrimer products. The use of microcrystalline copolymerized polymer structures in vitrimers offers improved properties such as self-healing and recyclability, making them an attractive alternative to traditional polymers.

The main conclusions of this study are as follows:

[1] The optimal conditions for obtaining the highest hydroxyl value of the glycolysate were using hard PU as waste, sodium hydroxide as the catalyst, and ethylene glycol as the solvent.
[2] The optimized glycolysate after distillation had a nearly transparent appearance.
[3] The optimal conditions for synthesizing vitrimers were HDI/total alcohol weight = 2, HDI trimer/total alcohol weight = 2, PEG/glycolysate = 0.5, and a reaction time of 60 min.
[4] The weight ratio of glycolysate to PEG was the most crucial factor affecting the self-healing rate of the product during synthesis.
[5] The elongation of the tensile strength test increased after modifying the experimental conditions.
[6] Future research could focus on further optimizing the synthesis conditions to improve the properties of the vitrimer products, as well as exploring potential applications in various industries. Additionally, investigations into the environmental impact of the synthesis process and the long-term stability of the products could also be beneficial.

Based on the results of this study, there are several suggestions for future research:

[1] Investigate the potential of using other waste materials, such as plastic bottles or packaging, as a feedstock for glycolysation product to synthesize vitrimers.
[2] Explore the use of other catalysts and solvents in the glycolysation process to optimize the hydroxyl value of the glycolysate.
[3] Further investigate the effects of different parameters, such as reaction temperature, on the synthesis of vitrimers to optimize their properties.
[4] Study the mechanical properties of the synthesized vitrimers in more detail, including their fatigue and creep behavior, to assess their suitability for practical applications.
[5] Investigate the potential of combining vitrimers with other materials, such as carbon fibers or nanoparticles, to further enhance their properties.

## Data Availability

The original contributions presented in the study are included in the article/Supplementary Material, further inquiries can be directed to the corresponding author.
